# Evaluation of the masking efficacy of caries infiltration in post-orthodontic initial caries lesions: 1-year follow-up

**DOI:** 10.1007/s00784-022-04843-w

**Published:** 2023-01-11

**Authors:** R. J. Wierichs, B. Abou-Ayash, C. Kobbe, M. Esteves-Oliveira, M. Wolf, I. Knaup, H. Meyer-Lueckel

**Affiliations:** 1 Department of Restorative, Preventive and Pediatric Dentistry, School of Dental Medicine, University of Bern, Freiburgstrasse 7, 3010 Bern, Switzerland; 2 Department of Orthodontics, University Hospital RWTH Aachen, Pauwelsstraße 30, 52074 Aachen, Germany

**Keywords:** Caries infiltration, Infiltrant, Post-orthodontic treatment, Enamel lesions, Esthetics, Resin, White spot lesion

## Abstract

**Objectives:**

This study aimed to qualitatively and quantitatively assess the masking efficacy and color stability of resin infiltration on post-orthodontic ICL after 1 year.

**Materials and methods:**

In 17 adolescents, 112 ICL (ICDAS-1: *n* = 1; ICDAS-2: *n* = 111) in 112 teeth were treated by resin infiltration (Icon, DMG) 3 to 12 months after bracket removal. The etching procedure was performed up to 3 times. Standardized digital images were taken before treatment (T_0_), 7 days (T_7_) and 12 months (T_365_) after treatment. Outcomes included the evaluation of the color differences between infiltrated and healthy enamel at T_0_, T_7_, and T_365_ by quantitative (colorimetric analysis (Δ*E*), ICDAS scores) and qualitative methods (5-point Likert scale (deteriorated (1), unchanged (2), improved, but not satisfying (3), improved and no further treatment required (4), completely masked (5)).) Differences between time points were analyzed by using Friedman test (Δ*Ε*) and chi-square tests (ICDAS).

**Results:**

The median color difference (25^th^/75^th^ percentiles) between carious and healthy enamel at baseline (Δ*Ε*_0_) was 10.2(7.7/13.6). A significant decrease was observed 7 days after treatment (Δ*Ε*_7_ = 3.1(1.8/5.0); *p* < 0.001; ICDAS; *p* < 0.001). No significant changes based on Δ*Ε* (*p* = 1.000), and ICDAS grade (*p* = 0.305) were observed between T_7_ and T_365_ (Δ*Ε*_12_ = 3.4 (1.8/4.9)). Furthermore, at T_365_ four experienced dentists classified 55% and 39% of the lesions as “improved and no further treatment required” and “completely masked,” respectively (Fleiss kappa: T_365_ = 0.851 (almost perfect)).

**Conclusion:**

Resin infiltration efficaciously masked post-orthodontic ICL 7 days and 12 months after treatment. These results for most of the teeth could not only be observed by quantitative but also by qualitative analysis.

**Clinical relevance:**

Resin infiltration efficaciously masks post-orthodontic initial carious lesions. The optical improvement can be observed directly after treatment and remains stable for at least 12 months.

**Supplementary Information:**

The online version contains supplementary material available at 10.1007/s00784-022-04843-w.

## Introduction

Initial caries lesions (ICL) can be considered as negative side effects of orthodontic treatment with fixed appliances and could be observed in up to 68.4% of orthodontically treated patients [1]. During orthodontic treatment fixed elements (e.g., brackets) may yield to a higher biofilm accumulation. As a consequence patients with higher cariogenic diet and impaired oral hygiene may develop caries lesions [2]. ICL develop quickly and are often an aesthetic burden to the patients even years after removal of the orthodontic appliances [3]. Consequently, a variety of non- or minimally invasive approaches have been suggested to avoid initiation, arrest the progression, and reverse the lesion or mask ICL during [4, 5] and after [6, 7] treatment with fixed appliances. However, most of these strategies do not seem to completely prevent the development or completely reverse ICL [5]. In most cases, the aesthetic appearance remains impaired [7].

After bracket removal ICL may superficially remineralize, since proper oral hygiene can be performed more effectively [8]. However, ICL being visible after six months or after are likely to persist and might be visually compromising, even when caries-preventive supplements (e.g., fluoride) are utilized [3, 8]. Therefore, two micro to minimally invasive treatments have been introduced for masking orthodontically induced ICL: Firstly, microabrasion, a technique based on mechanical removal of rather large amounts of the affected enamel [9]. By using this method optical appearance improves, generating a reduced and rough surface, which can yield to discoloration [9]. Secondly, resin infiltration, a less abrasive, and optically satisfying approach masks and seals ICL [6].

Sound enamel has a refractive index (RI) of 1.63. If the enamel surface is demineralized, intercrystalline spaces are then filled with air (RI = 1.00) or water (RI = 1.33). The overall RI of the demineralized area, thus, decreases and the surface appears chalky and dull [10]. By infiltrating a low viscosity resin (RI = 1.53) into these lesions’ overall RI of the infiltrated lesions increases and now getting closer to the RI of sound enamel. Light scattering is reduced, and the lesion optically resembles the surrounding healthy enamel and strengthens the micro-hardness of the enamel [11].

When resin infiltration is utilized for aesthetic reasons, firstly, predictability and, secondly, long-term color stability are major concerns [12]. Regarding predictability, the re-wetting test can be used to estimate the final masking effect [13]. A significant and strong correlation between the temporary masking effect during re-wetting and the final masking result after 7 days could be observed. However, regarding color stability, extrinsic discoloration and staining were observed in vitro [14, 15] and in vivo (Paris et al. 2010). In some studies, color differences of infiltrated lesions increased relatively to non-infiltrated demineralized or healthy surfaces after subsequent staining [14, 16]. Contrastingly, in other in vivo studies the masking effect of infiltrated ICL remained stable [6, 12, 17]. However, the follow-up period in most in vivo studies was only 6 months; only one study evaluated the masking effect after up to 2 years [18]. Thus, further in vivo studies evaluating the long-term masking efficacy of resin infiltration are needed [6].

Therefore, the present study aimed to qualitatively and quantitatively assess the masking efficacy and color stability of resin infiltration on post-orthodontic ICL after 1 year. The primary null hypothesis was that there are no significant differences in the colorimetric values between 7 days and 12 months after resin infiltration. The secondary null hypothesis was that there are no significant differences in colorimetric values between before resin infiltration and 7 days as well as 12 months after the treatment.

## Materials and methods

### Study design and patient selection

**Fig. 1:**
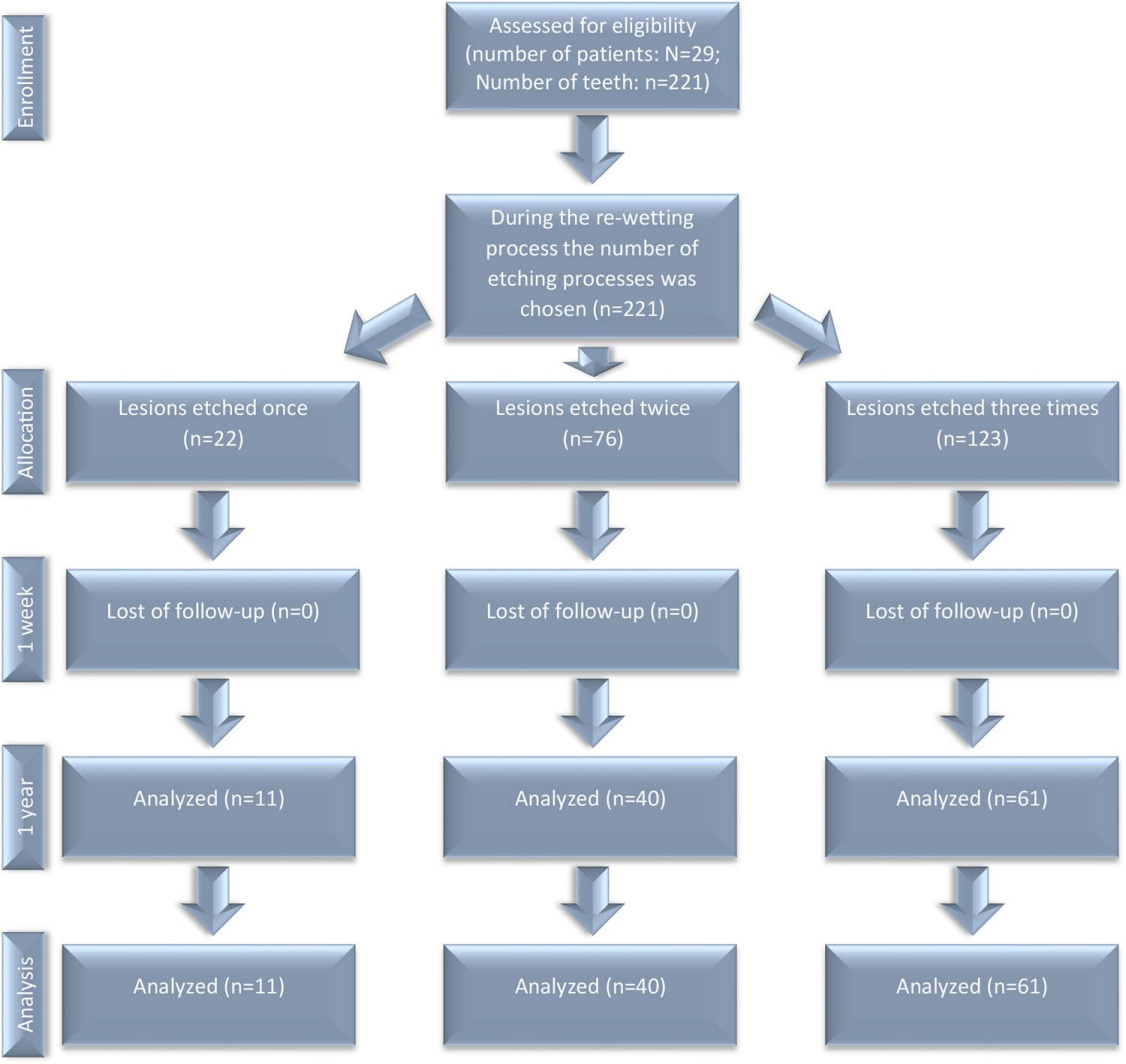
Study flow chart

The study was a clinical, single-center, prospective study (DRKS00005067). Approval was given by the ethical committee of the RWTH Aachen (EK 110/13). The study design has previously been described in detail and a selection of data referring re-wetting, quantitative analysis of light-induced fluorescence, and qualitative visual rating have been formerly published [13, 19]. Reporting follows the STROBE guideline for cohort studies [20]. All participants respectively their guardians provided their informed written consent. Based on the same cohort and cases 17 patients with 112 non-cavitated white spot lesions after removal of a fixed orthodontic appliance could be included in this 1-year follow-up (Fig. 1). ICDAS scores (International Caries Detection and Assessment System) were assigned and compared for every ICL at timepoints T0, T_7_, and T_365_. All treatments were performed in the Department of Orthodontics, RWTH Aachen University, Germany, not longer than 12 months after debonding of the fixed elements. Further exclusion criteria were allergies to the used materials and presence of cavitated lesions.

### Infiltration

Carious lesions were identified and distinguished from fluorosis by visualizing the typical rectangular form and sharp-edged demarcation caused by bonded brackets. Teeth were dried thoroughly for 10 s and ICDAS scores were evaluated subsequently. Affected teeth were then cleaned with a fluoride-free polishing paste (Cleanic; Kerr, Bioggio, Switzerland) and isolated with a liquid rubber dam (OpalDam; Ultradent, South Jordan, USA) to ensure gingival protection and reduce moisture. ICL were etched with 15% HCl gel (Icon etch; DMG, Hamburg, Germany) for 120 s. After rinsing with oil-free water for 30 s, the lesions were dried thoroughly with compressed air for 10 s and re-wetted with alcohol (Icon dry; DMG, Hamburg, Germany). The dentist then assessed the temporary masking effect of the lesion. In case of a subjectively unsatisfying result, the etching process was repeated. The lesions were then again etched, dried, and re-wetted up to a maximum of three times. Either after achieving a satisfying result or after the third and last etching process, the lesions were infiltrated according to the manufacturer’s recommendations. In brief, application of the Icon resin for 180 s, removing of all excessive material, subsequent light curing for 60 s, a second application of the resin for 60 s, removing of all excessive resin, subsequent light curing for again 60 s, and polishing with discs (Sof-Lex; 3 M, Saint Paul, USA) and a polishing bush (Occlubrush; Kerr, Orange, USA). All treatments were performed by one operator (C. K.)

### Photo documentation

**Fig. 2:**
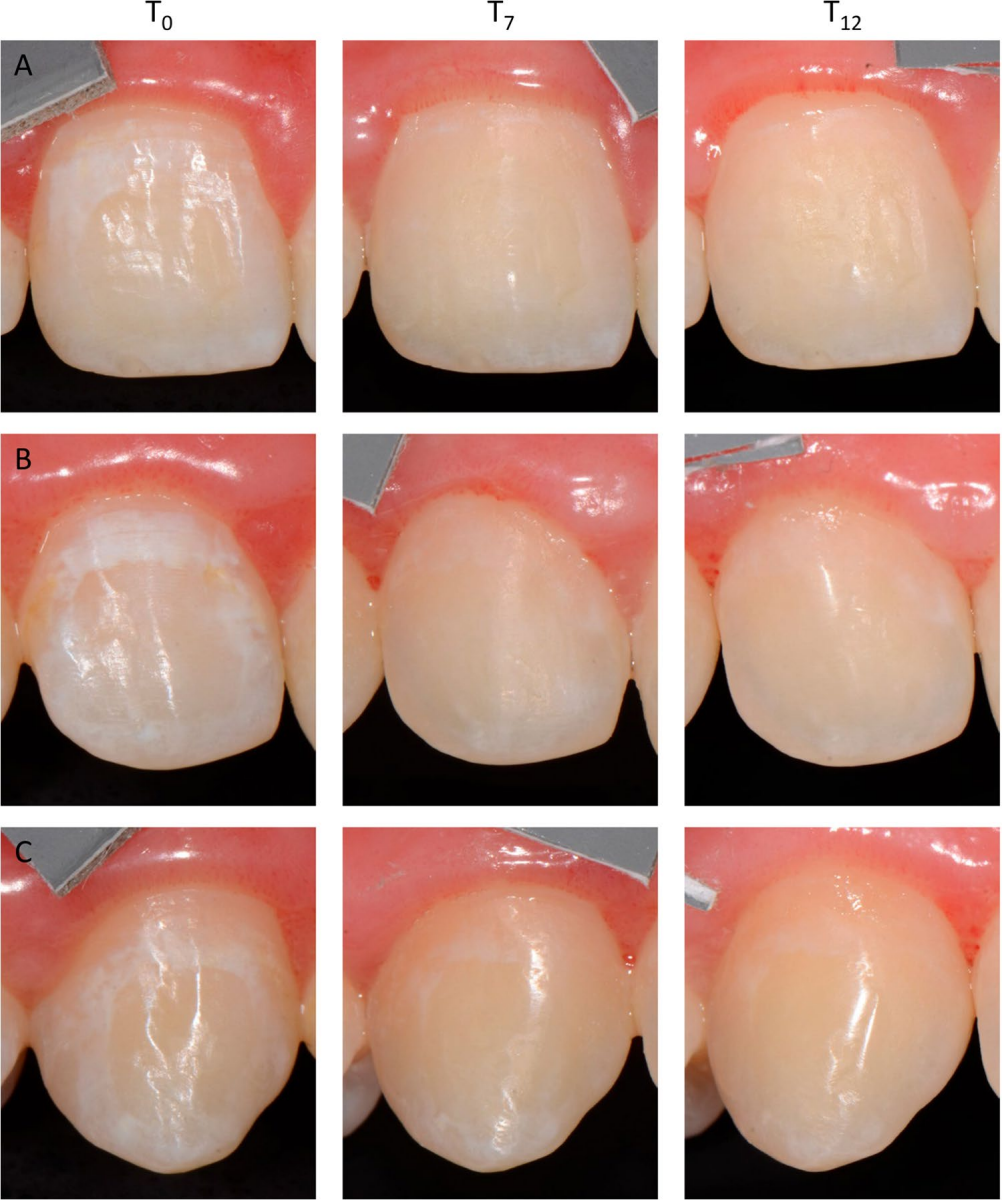
Teeth 11 (**A**), 12 (**B**), and 13 (**C**) showing distinct initial caries lesions at baseline (T0). Seven days (T7) and 12 months (T12) after treatment teeth 12 and 13 were classified as “improved and no further treatment required”; tooth 11 as “completely masked”

Digital, standardized single tooth and overall frontal photos were taken with a single-lens reflex camera (SLR) (Nikon D7000; Nikon, Chiyoda, Japan), a ring flash (Sigma EM-140 DG; Sigma, Kawasaki, Japan), and a macro lens (AF S Micro Nikkor 105 mm 1:2.8; Nikon, Chiyosa, Japan) before [T0] (baseline evaluation), 7 days [T_7_], and 12 months [T_365_] after the treatment (Fig. 2). All camera settings (1/250, aperture F29, iso-sensitivity 100 and a fixed white balance of 6250 K, ¼ left and right flash intensity) and the tooth-lens distances (20 cm) were standardized. All photos were taken by one operator (C. K.) for digital analysis and further standardization a gray card (18% gray; Mennon, Lake Forest, USA) was attached to the gingiva adjacent to the lesion.

### Colorimetric analysis

Digital color analysis and processing photoshop (Photoshop Adobe CS6; Adobe, San Jose, USA) were utilized. Color deviations in the photos were equalized by referring to the adjacent gray card. Four different measuring points (11 × 11 pixels) each were then set in carious (*c*) and adjacent healthy (*h*) enamel. Identical measuring points were chosen for the three different time points T0, T_7_, and T_365_. The L*a*b*-values of all measuring points were then documented in an excel sheet. Within the L*a*b color space tool, every color is defined by specific values of lightness (L*), green–red chromaticity (a*), and blue-yellow chromaticity (b*). The color differences between carious and healthy enamel (Δ*E*) as well as between different time points (Δ*E*) were calculated with the formula Δ*E*_*c*-*h*_ = ((*L*_*c*_ – *L*_*h*_)^2^ + (*a*_*c*_ – *a*_*h*_)^2^ + (*b*_*c*_– *b*_*h*_)^2^)^1/2^ [21] and e.g., Δ*E*_T1-T7_ = Δ*E*_T1_—Δ*E*_T7_ [14].

### Blinding

Due to the nature of the treatment procedure, neither the operator nor the patient could be blinded. However, outcome assessors and the statistician were blinded in relation to etching times (E1, E2, and E3)and picture time (T_7_ and T_365_).

### Qualitative visual analysis

The digital images of the three time points (T0, T7, and T_365_) were also used for visual assessment. Four trained operators (H.M-L., R.J.W., M.E.-O., B.A.-A.), with experiences in minimal-invasive and aesthetic treatments, performed a visual evaluation on tooth level (using teeth portraits). Prior to evaluation, all operators were calibrated by discussing clinical cases and agreeing on the degree of ICL expression and masking effect. All operators assessed the cases independently.

Using a 10-point Likert scale from 0 (no lesions visible) to 10 (the whole teeth is involved, high contrast, overall extension), the expression, extension, and contrast of the ICL on tooth level were evaluated. The success of treatment was also assessed by five categories: deteriorated (1), unchanged (2), improved, but not satisfying (3), improved and no further treatment required (4), completely masked (5).

### Statistical methods

Statistical analysis SPSS (SPSS Statistics 26; IBM, Armonk, USA) was utilized. A prospective power and sample size analysis were performed previously [13]. Furthermore, the retrospective power analysis for the smallest difference (difference between T0 and T_365_ for teeth being etched once) with 11 teeth still provided a power of at least 93% for Δ*E* (mean difference (SD): − 4.95 (4.19)).

The factors under evaluation were as follows:

- Time at three levels: (T0) situation before treatment, (T_7_) 7 days after treatment, (T_365_), 12 months after treatment
- Number of etching procedures: (*E*1) lesions etched once, (*E*2) lesions etched twice, (*E*_3_) lesions etched three times

Normal distribution was tested using the Shapiro–Wilk-test. Differences of Δ*E* in the different groups (E1, E2, and E3) were compared with the Kruskall-Wallis test with Bonferroni adjustment. Differences in Δ*E* between different time points (T0, T_7_, and T_365_) were analyzed using the Friedman test with Bonferroni adjustments. Differences in ICDAS values between different groups and between different time points were evaluated using chi-square tests. Qualitative visual scores mean values and standard deviations (SD) were used to describe the results of the 10-point Likert scale and the absolute number of scores were used to describe the results of the 5-point Likert scale [22]. The correlation between the qualitative and the quantitative evaluation (Δ*E*) were assessed using Spearman’s rank correlation [22]. The level of significance was set at 0.05.

## Results

### Study design and patient selection

Between November 2013 and December 2014, 29 patient (16 females, 13 male) with 221 lesions were included in this study. Due to non-appearance in the 1-year follow-up, 15 patients had to be excluded. Seventeen patients (7 females, 10 male) with a mean (SD) age of 16 (± 5.469) years at the first examination participated in this study. The 1-year follow-up examination took place between November 2014 and August 2015.

In total, 112 lesions (ICDAS 1 and 2) were treated in 112 teeth, of which 11 were upper jaw premolars, 61 upper jaw front teeth, 16 lower jaw premolars, and 24 lower jaw front teeth.

### Color differences ΔE

The median (25^th^/75^th^ percentiles) color difference between carious and healthy enamel at baseline (Δ*Ε*_0_) was 10.9 (8.2/13.2) regarding all 221 lesions (Δ*Ε*_0;221_) and 10.2 (7.7/13.6) regarding the 112 lesions (Δ*Ε*_0;112_) (Table 1). A significant decrease to Δ*Ε*_7;221_ = 4.0 (2.1/5.8) and Δ*Ε*_7;112_ = 3.1 (1.8/5.0), respectively, was observed 7 days after treatment (T_7_) (*p* < 0.001; Friedmann test). The 1-year follow-up-yielded stable values, no significant differences between Δ*Ε*_7;112_ and Δ*Ε*_365;112_ (3.1 (1.8/5.0)) (*p* = 0.725), but between Δ*Ε*_0;112_ and Δ*Ε*_365;112_ (*p* < 0.001) were observed.

**Table 1:** Median (25th /75th percentiles) color differences (∆E) between carious and healthy enamel at baseline, 7 days, and 12 months

Etching frequency	Time	*N*	Median	25^th^ percentile	75^th^ percentile	Significance
Overall	**T**_**0**_**—baseline**	112	10.2	7.7	13.6	Reference
	**T**_**7**_**—7d**	101	3.1	1.8	5.0	< 0.001
	**T**_**365**_—**12 M**	112	3.4	1.8	4.9	< 0.001
Once	**T**_**0**_**—baseline**	11	7.0	5.6	10.9	Reference
	**T**_**7**_**—7d**	9	1.5	1.0	1.8	0.002
	**T**_**365**_**—12 M**	11	1.7	0.9	3.2	0.010
Twice	**T**_**0**_**—baseline**	40	9.7	7.4	13.5	Reference
	**7d T**_**7**_**—7d**	39	2.1	1.4	4.0	< 0.001
	**T**_**365**_**—12 M**	40	3.0	1.9	4.3	< 0.001
Three times	**T**_**0**_**—baseline**	61	11.6	8.7	14.0	Reference
	**T**_**7**_**—7d**	53	4.7	2.8	6.0	< 0.001
	**undefined T356 - 12 M**	61	4.4	2.4	5.8	< 0.001

*P*-values indicate (statistically) significant differences in Δ*E* between T_0_ and T_7_ as well as T_0_ and T_365_ (Friedman test with Bonferroni adjustments). No significant difference could be observed between T_7_ and T_365_ (*p* = 1.000)

The results did not change when analysis of different time points was done separately for the different number of etching procedures (Table 1).

### ICDAS scores

At baseline (T0) one lesion was scored as ICDAS 1 and 111 as ICDAS 2 (Table 2). The ICDAS scores significantly decreased seven days after resin infiltration (*p* < 0.001; chi-square test). Twelve months after treatment ICDAS scores showed no further change compared to T_7_ (*p* = 0.355; chi-square test). Lesions still ranged from ICDAS 0 to 2. Fifty-six lesions were scored as ICDAS 0, 11 as ICDAS 1, and 38 as ICDAS 2.

**Table 2:** ICDAS scores with different etching frequencies at baseline, 7 days, and 12 months

Etching frequency	Time	*N*	0	1	2	Significance
Overall	**T**_**0**_**—baseline**	112	0	1	111	Reference
	**T**_**7**_**—7d**	101	47	11	43	< 0.001
	T_365_—**12 M**	112	63	11	38	< 0.001
Once	**T**_**0**_**—baseline**	11	0	0	11	Reference
	**T**_**7**_**—7d**	9	4	5	0	< 0.001
	T_365_—**12 M**	11	8	3	0	< 0.001
Twice	**T**_**0**_**—baseline**	40	0	0	40	Reference
	**T**_**7**_**—7d**	39	31	3	5	< 0.001
	T_365_—**12 M**	40	34	1	5	< 0.001
Three times	**T**_**0**_**—baseline**	61	0	1	60	Reference
	**T**_**7**_**—7d**	53	12	3	38	< 0.001
	**T**_**365**_**—12 M**	61	21	7	33	< 0.001

*P-*values indicate (statistically) significant differences in ICDAS scores between T_0_ and T_7_ as well as T_0_ and T_365_ (chi-square tests). No significant difference could be observed between T_7_ and T_356_ (*p* ≥ 0.145)

### Qualitative visual analysis

The severity of aesthetic impairment due to white spot lesions was rated using the 10-point Likert scale. At baseline (T0), the ICL were rated with an average of 3.7 points (SD 1), indicating that mild-to-moderate cases have been included in the present study. One year after treatment (T_365_), the ICL were rated with an average of 0.9 points (SD 0.9) (inter-observer reliability; Fleiss kappa: T_0_: 0.440 (moderate agreement); T_365_: 0.769 (substantial agreement)).

The optical improvement could also be seen in the 5-point Likert scale. At T_365_, the results of only one tooth were classified as unchanged, whereas 55% and 39% of the results were classified as improved and no further treatment required and completely masked, respectively (Fleiss kappa: T_365_: 0.851 (almost perfect)).

### Correlation analysis

A significant and moderate to strong correlation was observed between the qualitative (10-point Likert scale) and the quantitative evaluation (Δ*E*) on tooth level (T0: *r* = 0.508; *p* < 0.001; T_7_: *r* = 0.791; *p* < 0.001; T_365_: *r* = 0.556; *p* < 0.001).

A significant and moderate to strong correlation was also observed between the change in the quantitative evaluation (5-point Likert scale) and the change in colorimetric values (after 7 days (ΔE_7_) and 12 month (ΔE_356_)) (T_7_: *r* =  − 0.761; *p* < 0.001; T_365_: *r* =  − 0.528; *p* < 0.001).

### Adverse effects

No adverse or side effects were recorded during the follow-up period.

## Discussion

The present study investigated the masking efficacy of resin infiltration qualitatively and quantitatively 12 months after its application. We confirmed that resin infiltration efficaciously masks post-orthodontic initial carious lesions for at least twelve months. A significant reduction of the colorimetric values, ICDAS scores, and visual impairment could be observed directly after treatment. Results remained stable at the 12 months follow-up. Therefore, the primary null hypothesis could be confirmed. Furthermore, the secondary null hypothesis had to be rejected since there are differences in colorimetric values between before resin treatment and the time points seven days respectively 12 months after resin infiltration.

The present study use of resin infiltration significantly decreased ∆*E*-values. This is in alignment with recent in vivo studies [7, 17, 23, 24]. By using a crystal eye spectrometer [7, 17, 24] or digital photographs [18, 23, 25], the colorimetric analyses showed resin infiltration to significantly mask (post-orthodontic) initial caries lesions. However, final ∆*E*-values differed possibly due to the varying depths of the lesions and/or the varying etching protocols, ranging only etching once [7, 23, 25] to up to three times as in the present study, depending on the depth and the visibility of the lesion [17, 18]. Nonetheless, ∆*E*-values in all studies decreased immediately after treatment and remained stable for 7 days [25], 6 months [7], 12 months [17], or 24 months [18].

Qualitatively 55% and 39% of the lesions were classified as “improved and no further treatment required” and “completely masked,” respectively. Interestingly, the qualitative outcome showed a significant, moderate to strong correlation with the quantitative analysis (Δ*E*). This is in line with a recent study in which a significant and strong correlation between quantitative and qualitative results before and directly after infiltration could be observed [23].

No untreated control group was included in the present study. The absence of an untreated control group is one of the major limitations of this 1-year follow-up, since ICL tend to regress within the first 3 to 6 months after debonding [8, 26]. Although the authors of a recent study raised the question of whether a split-mouth design is ethically feasible at all, when analyzing infiltration[18] a parallel-group design could, of course, have been used. However, due to the convincing evidence of the masking efficacy of resin infiltration, we omitted this.

A second limitation of this study is that the duration between the debonding of the brackets and infiltration of the ICL varied between 3 and 12 months (mean time (SD) after debonding 3 (3) months). The minimum interval after bracket removal has been considered to attain at least 3 months to allow ICL to remineralize (superficially) and possibly regress also aesthetically before treatment [26]. With longer intervals between debonding and infiltration, a thicker surface layer can be assumed, impeding penetration of the infiltrant, and consequently resulting in large numbers of unfavorable aesthetic results. Although this interval was not standardized, we considered debonding times that were longer than 12 months ago as an exclusion criterion.

The primary publication addressed the question of effects of one, two, or three etching procedures on lesions scored as ICDAS 2 [13]. It could be observed that the number of etching procedures significantly correlated with the baseline Δ*E*. More obvious lesions received more likely two or three etching procedures. Consequently, it would be interesting to see if the number of etching steps can be varied between ICL with different ICDAS scores. Since in the present study lCL were scored as ICDAS 2 (except one lesion), this cannot be answered within the present study. Furthermore, recent studies suggests that the time interval between bracket removal and resin infiltration appears to play an important role for the successful masking of ICL since remineralization is not yet completed and the surface is softer and therefore easier to penetrate [12]. To answer this question, we are running a study to evaluate, firstly, the masking results in ICL that were infiltrated during treatment with fixed orthodontic appliances and, secondly, in the future, to compare these results to ICL that were initially fluoridated and will be infiltrated after fixed appliances removal [27]. However, there are no long-term results yet due to the recent start.

Long-term color stability was assessed by using different methods: colorimetric analysis by using the L*a*b*-values, ICDAS score and by qualitative visual assessment. For all outcomes, no statistically significant differences between the values 7 days and 12 months after infiltration could be observed. This is in line with recent studies presenting follow-up periods of 6 [7], 12 [17], and 24 months[18]. The latter study even showing stable results up to 45 months [18]. Consequently, resin infiltration seems to be a suitable method for a long-term aesthetical improvement of ICL.

Based on our results, it can be corroborated that resin infiltration efficaciously masks post-orthodontic initial caries lesions immediately. Moreover, 1 year after significant reduction of Δ*E* remained just slightly below the threshold for perception. Color stability could also be confirmed by significantly decreased ICDAS scores that also remained unchanged during the follow-up period. Quantitative and qualitative assessment showed good to substantial correlations.

## Supplementary Information

Below is the link to the electronic supplementary material.

Supplementary file1 (DOCX 35 KB)

## Data Availability

All data generated or analyzed during this study are included in this article [and/or] its supplementary material files. Further enquiries can be directed to the corresponding author.
